# Rapid progression and future of environmental DNA research

**DOI:** 10.1038/s42003-019-0330-9

**Published:** 2019-02-27

**Authors:** Mathew Seymour

**Affiliations:** Molecular Ecology and Fisheries Genetics Laboratory, School of Natural Sciences, Bangor University, Bangor, Gwynedd, LL57 2UW UK

## Abstract

Environmental DNA based research is a new field within molecular ecology that is seeing an amazing increase in research activity. In our *Communications Biology* article, we studied the degradation of eDNA in variable systems. Presented here is a short overview of eDNA science and current research activities underway in North Wales.

## Abstract (web summary)

Mathew Seymour discusses the current status of using environmental DNA derived directly from natural environments to study biodiversity, and its applications in conservation and ecological research. In his Comment, he explores how eDNA as a technology can foster multi-disciplinary collaboration.

## Environmental DNA for assessing biodiversity

Scientific advancement is periodically stimulated by key developments that lead to expedited research efforts. One such development, the discovery of DNA, has drastically altered scientific thought leading to many major advancements and creation of several fields of study within medicine, agriculture, forensics, evolution and molecular ecology. The field of molecular ecology, which uses genetic methods to address ecological questions, has recently seen an explosion of scientific activity surrounding the development and use of environmental DNA (eDNA).

Environmental DNA, in its simplest sense, is DNA extracted from any type of environmental sample (e.g. soil, water, air, etc.), without isolation of a particular organism^1,2^. Combined with modern genetic tools, eDNA offers a non-invasive means to identify species or communities associated with the environment from which the DNA was extracted. Since its emergence as a reliable tool for conservation and invasion biology^3^, the number of eDNA studies published has exponentially increased, many government agencies have established several eDNA-based monitoring programs, and a plethora of eDNA service start-up companies have been created. Moreover, as the excitement and increased focus on eDNA brings more researchers into the fold, more scientific questions, criticisms and innovations begin to unfold. While innovations are the propellant for scientific advancement and economic and social progress, limitations for using eDNA effectively are becoming increasingly apparent and will need to be addressed for eDNA research to be an established facet of molecular lexicon into the future. Our recent work in *Communications Biology*^4^, and the subsequent work stemming from these findings, will assist in answering some key questions with regards to the unknown ecology of eDNA and its use as a biodiversity monitoring tool.

Unlike traditional biodiversity assessment methods, where captured or recorded individuals are used to determine presence or abundance, eDNA-based biodiversity assessment relies on our ability to capture the genetic signature left behind by organisms through shedding, excreting, decaying, etc. Environmental DNA based research is dependent on our capacity to accurately match the left-behind genetic signature to the correct species. Several studies have already successfully shown eDNA can be a highly accurate biomonitoring tool, more so than traditional methods in several instances^5–7^_._ Originally, studies roughly assumed eDNA behaved similarly across broad environmental sample types, such as soil, water and pollen. However, researchers soon realized that eDNA findings and interpretations are not only highly variable across environmental sample types, but also across ecological systems when using the same type of environmental sample^8,9^. For example, eDNA extracted from water samples is relatively short lived (days), compared to soil or ice (years), which makes water eDNA a good candidate for biomonitoring since the signal represents the presence of recent species. However, as studies have pointed out, the spatial and temporal extent represented by a water-based eDNA sample is dependent on local environmental factors including water transport dynamics and abiotic and physical conditions^4,9–11^.

Recently, we looked to experimentally assess the transport and degradation dynamics of eDNA in river systems using a set of field based artificial streams, designed at Cardiff University^12^. Our findings show that for multiple species, eDNA degrades over time in a logistic manner with degradation being further exasperated by acidic conditions^4^. Additionally, we determined that the transport time of eDNA is extensive in rivers, with signals detectable up to 32 km. Our findings have important implications for ongoing and future eDNA research looking to advance the ecological understanding and application of eDNA-based methods within molecular ecology and the eminent extension into additional fields of research.

## The evolution of eDNA studies

Presently, eDNA studies can be categorised into two main groups: targeted (species-specific) and semi-targeted (community) approaches. Both categories are often discussed simultaneously but differ drastically in their methodology, interpretations and accuracy. Species-specific studies use assays tailored to particular species to target specific DNA fragments in an environmental sample. Studies may focus on a single species or utilize a set of assays to use on a single environmental sample. Presence or absence of the targeted DNA, as in the classical eDNA studies, is still conducted using standard PCR, though quantification is becoming more standard using quantitative PCR or digital PCR chemistry. Species-specific approaches, when designed stringently and thoroughly validated, can be a highly reliable eDNA based method^13^, and often effectively linked to biomass and abundance of the target organism^14^.The original usage of targeted eDNA, which is often attributed to the rise in eDNA research, was the detection of the invasive American bullfrog in France^15^. Eventually, eDNA was put under intense scrutiny surrounding the establishment of eDNA as part of the Asian carp monitoring efforts in the United States^16^. Recently more government-supported eDNA monitoring programs and assay developments have arisen for a wide range of invasive and conservation status species. As such, a majority of species-specific eDNA research is focused on designing and standardizing assays for conservation and invasion management. However, as our work and several others points out, the abiotic and spatial-temporal dynamics of eDNA are not constant for all environments, and greater effort will be needed to ensure policies take environmental conditions into account when interpreting eDNA results. Recent efforts into substrate absorption effects^17^ and additional abiotic effects on degradation^8^ combined with spatial modelling of eDNA across river networks^18^ are all promising avenues in disentangling the intricate details of eDNA ecological dynamics. While developments and advancements continue to progress the frontier of species-specific eDNA research, there is far greater scientific potential being unveiled within the realm of community eDNA studies, which is beginning to open the door to multi-disciplinary studies and a new wave of discovery science.

An overarching aim of the research community is to use eDNA as a means to identify and summarize the biological communities, much like a tricorder in star trek^19^. While the molecular methodology for targeted eDNA studies are relatively consistent across studies, community-based methods encompass a wide range of high-throughput sequencing techniques including, metabarcoding, long-read, shot-gun mitogenomics, genome skimming, etc. Simplistically, community-based eDNA analyses look to associate all available DNA strands in an environmental to their species of origin. Accurate identification of sequences to species is highly reliant on existing genetic databases to associate the sequenced data to the appropriate taxonomic origin, which are mostly incomplete for any given study, often leading to course taxonomic assignment. Despite the present limitations in taxonomic identification resolution, community-based eDNA research is the forefront of eDNA research efforts, with several government agencies and companies vying to successfully develop reliable pipelines for community based assessment, particularly for the current major organism groups used for environmental assessment^20^.

The potential for inter-disciplinary research is also much higher with community-based eDNA (compared to species-specific) as investigation of complimentary matrix datasets can be used to infer ecological networks^21^, test ecological theory^22^, assess eco-toxicology dynamics^23^, etc. Additionally, assessing the functional and chemical diversity of environmental samples (e.g. metabolomics and transcriptomics) in conjunction with high throughput taxonomic identification, opens up further potential for a new era of cross-domain discovery-based science and hypothesis testing, including the possibilities of new sets of environmental indicator groups. However, present studies are still testing the application of community-based eDNA approaches in natural environments, with several recent studies supporting findings from mock community tests, which is essential for showing the reliability of the methods^5,7^.

## Environmental DNA in lotic ecosystems

Our present understanding of eDNA dynamics is still largely limited in space and time, but forthcoming research efforts are now looking to rigorously test natural eDNA community dynamics and eventual machine-assisted learning discovery, including the work currently underway by the National Environment Research Council (NERC)-funded LOFRESH project.

Seymour et al (2018) is a small part of the LOFRESH project, which is a four-year NERC (UK) funded consortium that began in 2016, with the overarching aim to understand the ecological relevance of eDNA in lotic (e.g. rivers) environments (http://lofresh.bangor.ac.uk/). Our experimental findings have laid the groundwork regarding the degradation and transport dynamics of eDNA, while our present efforts are robustly assessing lotic community eDNA temporal, spatial and environmental dynamics across the Conwy river drainage in North Wales. Using a combination of aquatic macroinvertebrate, chironomid exuvia and eDNA sampling methods, we conducted sampling every three weeks starting in April 2017 for 15 sites along a 35 km stretch of the Conwy river, concluding in April 2018. In conjunction we also seasonally sampled macroinvertebrates and eDNA from 14 tributary sites across the Conwy drainage representing a wide range of landuse types, including agriculture, moorlands, broadleaf forest, acid grasslands and urbanized environments, for which long term chemistry data has been collected by our collaborators at the Centre of Ecology and Hydrology (CEH). On a wider scale, we have also collaborated with CEH to conduct a joint traditional and eDNA-based Wales-wide environmental assessment to further test eDNA community dynamics across multiple ecosystems. After preliminary optimization and selection, we have created metabarcode libraries that semi-target macroinvertebrate, fish, diatom and universal (18 S) communities for all collected samples. Presently, in collaboration with the University of Birmingham, we are sequencing and bioinformatically processing the collected data to present to the wider scientific community shortly. While these data will initially be used to answer relatively basic, but essential, questions regarding the spatial and temporal dynamics of lotic eDNA, they will also represent one of the largest eDNA based datasets. The size of the collected data will allow for a wide range of ecological, bioinformatics and molecular questions to be assessed and explored further.

## Conclusions

Environmental DNA has provided a catalyst for an amazing wave of research. Combined with advances in sequencing technologies, computer-assisted learning and chemical analyses we are potentially facing a renaissance in biological science, most assuredly within the realm of molecular ecology. The impact that eDNA has already had on all aspects of relevant research, despite its recent development, is astounding and will undoubtedly continue to influence careers and policies for years to come, with all manner of mistakes and updates along the way. It will be paramount to ensure collaboration is maintained within and among investigation bodies and to put effort into conveying the importance of such findings to the public and managers. The frontiers of eDNA research—particularly the semi-targeted community approach—are vast, but the potential is enormous with applications across all areas of science, including and perhaps even more so to traditional research thinking. As with all realms of academia, multi-disciplinary collaboration is essential, and embracing the unknown with curiosity and teamwork, as with the LOFRESH group, is where pushing the boundaries of science moves from proposal to reality.

## References

[1] Taberlet, P., Bonin, A., Zinger, L. & Coissac, E. *Environmental DNA: For Biodiversity Research and Monitoring*. (Oxford University Press, Oxford, UK, 2018).

[2] Deiner, K. et al. Environmental DNA metabarcoding: Transforming how we survey animal and plant communities. *Mol. Ecol*. **26**, 5872–5895 (2017). 10.1111/mec.14350 28921802

[3] Goldberg CS, Strickler KM, Pilliod DS (2015). Moving environmental DNA methods from concept to practice for monitoring aquatic macroorganisms. Biol. Conserv..

[4] Seymour M (2018). Acidity promotes degradation of multi-species environmental DNA in lotic mesocosms. Commun. Biol..

[5] Valentini A (2016). Next-generation monitoring of aquatic biodiversity using environmental DNA metabarcoding. Mol. Ecol..

[6] Mächler E, Deiner K, Steinmann P, Altermatt F (2014). Utility of environmental DNA for monitoring rare and indicator macroinvertebrate species. Freshw. Sci..

[7] Bista I (2017). Annual time-series analysis of aqueous eDNA reveals ecologically relevant dynamics of lake ecosystem biodiversity. Nat. Commun..

[8] Collins RA (2018). Persistence of environmental DNA in marine systems. Commun. Biol..

[9] Barnes MA, Turner CR (2016). The ecology of environmental DNA and implications for conservation genetics. Conserv. Genet..

[10] Deiner K, Altermatt F (2014). Transport distance of invertebrate environmental DNA in a natural river. PLoS One.

[11] Jerde CL (2016). Influence of stream bottom substrate on retention and transport of vertebrate environmental DNA. Environ. Sci. Technol..

[12] Durance I (2016). The challenges of linking ecosystem services to biodiversity. Adv. Ecol. Res..

[13] Goldberg CS (2016). Critical considerations for the application of environmental DNA methods to detect aquatic species. Methods Ecol. Evol..

[14] Doi H (2015). Use of droplet digital PCR for estimation of fish abundance and biomass in environmental DNA surveys. PLoS One.

[15] Ficetola GF, Miaud C, Pompanon F, Taberlet P (2008). Species detection using environmental DNA from water samples. Biol. Lett..

[16] Jerde CL (2013). Detection of Asian carp DNA as part of a Great Lakes basin-wide surveillance program. Can. J. Fish. Aquat. Sci..

[17] Shogren AJ (2017). Controls on eDNA movement in streams: transport, retention, and resuspension. Sci. Rep..

[18] Carraro L, Hartikainen H, Jokela J, Bertuzzo E, Rinaldo A (2018). Estimating species distribution and abundance in river networks using environmental DNA. Proc. Natl Acad. Sci. USA.

[19] Wikipedia Contributors. Tricorder. Wikipedia, The Free Encyclopedia. https://en.wikipedia.org/w/index.php?title=Tricorder&oldid=869166423 (2018).

[20] Pawlowski J (2018). The future of biotic indices in the ecogenomic era: Integrating (e)DNA metabarcoding in biological assessment of aquatic ecosystems. Sci. Total Environ..

[21] Bohan DA (2017). Next-generation global biomonitoring: large-scale, automated reconstruction of ecological networks. Trends Ecol. Evol..

[22] Yoccoz NG (2012). The future of environmental DNA in ecology. Mol. Ecol..

[23] Prosser JI (2015). Dispersing misconceptions and identifying opportunities for the use of omics in soil microbial ecology. Nat. Rev. Microbiol..

